# Implementation of the European Society of Cardiology Zero/Two-Hour High-Sensitivity Troponin Pathway for Non-ST-Elevation Myocardial Infarction Diagnosis: A Real-World Observational Study From a UK District Hospital

**DOI:** 10.7759/cureus.94266

**Published:** 2025-10-10

**Authors:** Mohamed Haggag, Ahmed Shehabeldein

**Affiliations:** 1 Respiratory Medicine, Blackpool Teaching Hospitals NHS Foundation Trust, Blackpool, GBR; 2 General Medicine/Acute Medicine, Blackpool Victoria Hospital, Blackpool, GBR; 3 General Medicine, Blackpool Victoria Hospital, Blackpool, GBR

**Keywords:** clinical adherence, diagnostic pathway, esc guidelines, high-sensitivity troponin, nstemi

## Abstract

Background

High-sensitivity cardiac troponin (hs-cTn) assays enable accelerated diagnostic pathways for non-ST-elevation myocardial infarction (NSTEMI). The 2023 European Society of Cardiology (ESC) guidelines recommend zero/one-hour or zero/two-hour protocols, but real-world adherence remains inconsistent. This study evaluated the impact of structured pathway implementation on diagnostic efficiency, test utilisation, and safety outcomes in a UK district general hospital.

Methodology

We conducted a retrospective, pre-post, observational study including adults presenting with chest pain who underwent hs-cTn testing. Data were collected during two periods: November 2024 (pre-implementation cohort, n = 595) and June 2025 (post-implementation cohort, n = 340). Between cohorts, interventions included targeted staff education, visual algorithm prompts, and collaboration with laboratory services. Outcomes included adherence to the ESC zero/two-hour pathway, unnecessary repeat testing, inappropriate testing in non-cardiac presentations, time to troponin sampling, length of stay, and documentation of risk stratification.

Results

Adherence to the ESC zero/two-hour pathway improved from 74.2% to 94.4% (p < 0.001). Unnecessary repeat testing in patients with very low initial troponin decreased from 24.6% to 4.4%. Efficiency improved significantly: time to first hs-cTn reduced by 13.5 minutes, zero to two-hour testing interval by 133.8 minutes, and length of stay by 313 minutes (all p < 0.001). Documentation of the ESC pathway (1.0% vs. 74.1%) and History, Electrocardiogram, Age, Risk factors, and Troponin (HEART) score use (1.0% vs. 62.8%) increased markedly. No rise in missed NSTEMI or 30-day major adverse cardiac events was observed. However, >70% of hs-cTn requests remained for non-cardiac presentations.

Conclusions

Structured implementation of the ESC zero/two-hour hs-cTn pathway substantially improved diagnostic efficiency, adherence, and safety without increasing adverse outcomes. Persistent overuse of hs-cTn in non-cardiac presentations highlights the need for system-level stewardship. The observed improvements in diagnostic speed and reduced length of stay also translate into significant operational benefits, including increased bed availability and improved patient flow, without compromising patient safety. Future efforts will focus on digital integration and continuous audit-feedback systems to reinforce adherence and address inappropriate troponin use in non-cardiac presentations.

## Introduction

One of the most common presenting complaints in emergency departments and acute medical units is chest pain. Rapid and accurate evaluation is essential to rule in or rule out acute coronary syndromes, particularly non-ST-elevation myocardial infarction (NSTEMI), which can represent a challenge [1]. High-sensitivity cardiac troponin (hs-cTn) assays have significantly reshaped the diagnosis of myocardial infarction by promoting earlier and more reliable detection of myocardial injury [2].

The European Society of Cardiology (ESC) 2023 guidelines recommend accelerated diagnostic protocols using either the zero/one-hour or zero/two-hour algorithm for assessing patients with suspected NSTEMI [3]. Both algorithms are based on serial hs-cTn measurements and have demonstrated significantly high sensitivity and negative predictive value, allowing healthcare providers to confidently and safely rule in or rule out myocardial infarction within a short time frame [4,5]. The zero/one-hour algorithm is preferred in most settings due to its speed, while the zero/two-hour algorithm offers a validated alternative where logistics or staffing make earlier testing less feasible.

Despite their proven clinical utility, adherence to these algorithms remains inconsistent in real-world practice [6]. Common challenges include unnecessary repeat testing, hs-cTn ordering in patients without suggestive cardiac symptoms, and confusion over assay-specific thresholds. Such deviations not only increase healthcare costs and length of stay but may also delay appropriate care or lead to missed diagnoses [7]. Furthermore, excessive reliance on troponin testing in non-cardiac presentations may contribute to diagnostic uncertainty and resource misuse [8].

This study was conducted at a large district general hospital in the United Kingdom to evaluate the implementation of the ESC hs-cTn zero/two-hour algorithm and assess its impact on diagnostic efficiency, test utilisation, and clinical safety, providing real-world evidence to inform broader adoption strategies, given that data were derived from the actual delivery of care. We hypothesised that implementing the zero/two-hour algorithm would improve diagnostic efficiency by enabling earlier rule-in or rule-out of NSTEMI, reducing unnecessary repeat troponin testing, and maintaining patient safety with low rates of missed myocardial infarction.

## Materials and methods

Study design

**Figure 1:**
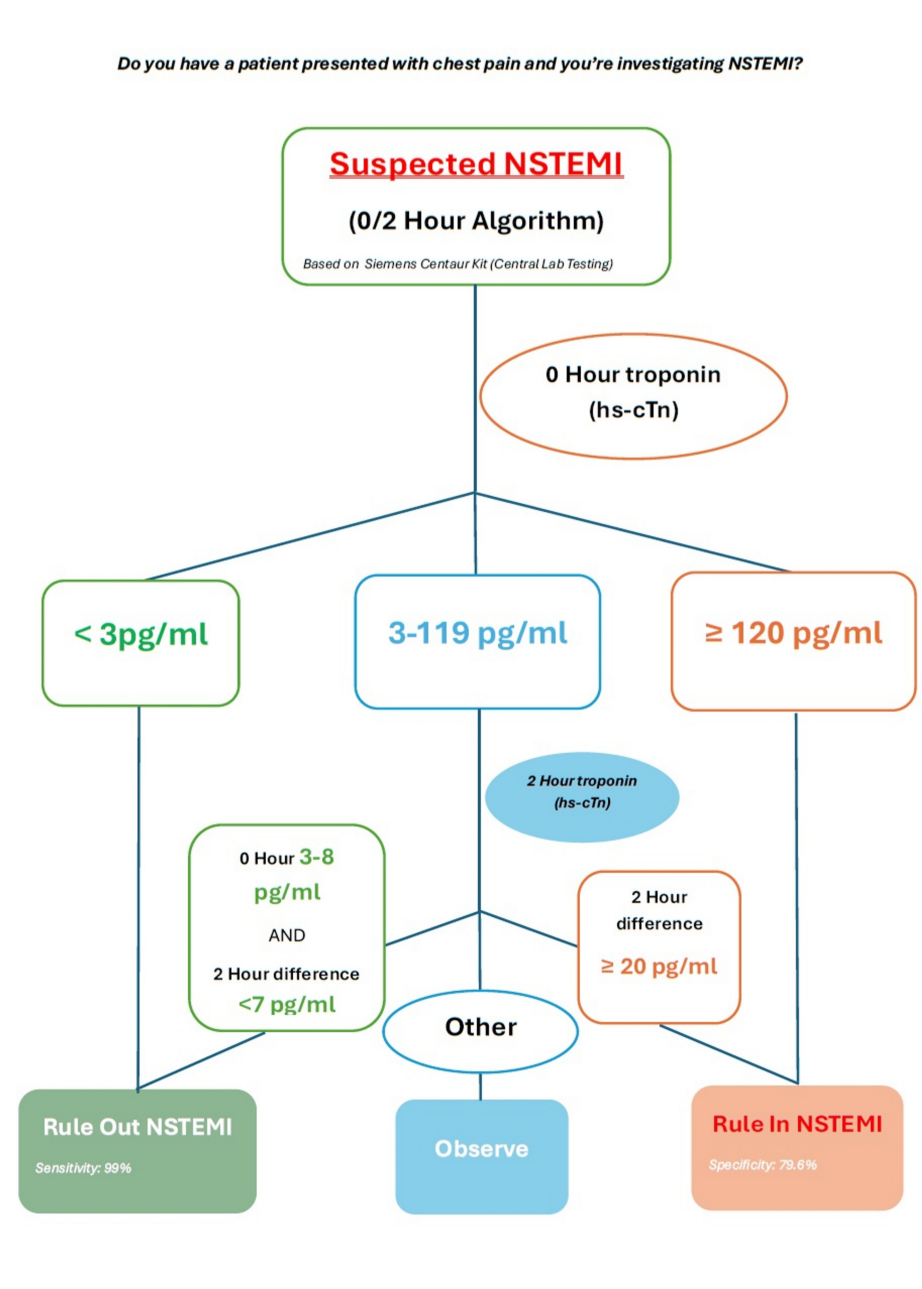
NSTEMI zero/two-hour rule-in/out algorithm based on the Siemens Centaur® high-sensitivity troponin I assay. NSTEMI: non-ST-elevation myocardial infarction; hs-cTn: high-sensitivity cardiac troponin

This was a retrospective, observational cohort study using a pre-post implementation design. Adult patients presenting with suspected cardiac chest pain who underwent high-sensitivity troponin testing were evaluated in two cohorts: a pre-implementation cohort (November 2024) and a post-implementation cohort (June 2025). Outcomes were compared to assess the impact of structured implementation of the ESC zero/two-hour pathway. The study was conducted at Blackpool Victoria Hospital, a district general hospital in the United Kingdom. Between the two cohorts, targeted interventions were introduced, which involved educational sessions for foundation doctors, resident doctors, nurse practitioners, and staff nurses; display of simplified visual aids and algorithm posters (Figure 1) in high-traffic clinical areas (Emergency Department, Same-Day Emergency Care (SDEC) unit, and Acute Medical Unit); and collaboration with the central laboratory to standardise interpretation of hs-cTn thresholds [9].

Study period and population

Pre-implementation cohort data were collected from November 1 to November 30, 2024. Post-implementation cohort data were collected from June 1 to June 30, 2025. Adults who were 18 years or older, presented with chest pain suggestive of cardiac ischemia, and had a zero-hour hs-cTn test using the Siemens Centaur® high-sensitivity troponin I assay were included in the study. The manufacturer-reported analytical characteristics for this assay were as follows: limit of blank: 0.5 ng/L; limit of detection: 1.6 ng/L; and limit of quantification (coefficient of variation = 20%) of 2.5 ng/L with serum being an accepted sample type. Exclusion criteria were patients with ST-elevation myocardial infarction or unstable angina (not requiring troponin testing).

Initial hs-cTn results were stratified into the categories shown in Table 1. These thresholds were defined according to the ESC 2023 guidelines and assay-specific recommendations [2,3].

**Table 1:** Troponin categories.

Category	Level
Very low	<3 ng/L
Low	3–119 ng/L
High	≥120 ng/L

Outcome measures

The primary outcomes measured included the adherence to the ESC zero/two-hour algorithm, the proportion of unnecessary repeat testing in patients with very low initial troponin (<3 ng/L), and the proportion of troponin tests ordered for patients without chest pain. Inappropriate hs-cTn ordering was defined as ordering the test for patients who did not present with chest pain or other symptoms suggestive of acute coronary syndrome. These measures were selected to evaluate the clinical appropriateness and diagnostic efficiency of hs-cTn testing. Secondary outcomes included recording of the HEART score (History, ECG, Age, Risk Factors, and Troponin) aiding in the six-week risk assessment of major adverse cardiac events (MACE) in patients with chest pain, length of stay duration, safety outcome measures as missed NSTEMI defined as low troponin in context of chest pain, where troponin test was needed but not done as deemed low, later verified by further hospital admission with high troponin and coronary angiography confirming coronary artery disease, and 30-day MACE, including the composite of total death, MI, stroke, hospitalization because of heart failure, and revascularization, including percutaneous coronary intervention, and coronary artery bypass graft. Unnecessary repeat troponin testing was operationally defined as any repeat hs-cTn measurement performed within the zero/two-hour algorithm for patients who had an initial hs-cTn level <3 ng/L (very low) and no other clinical features suggestive of ongoing ischemia or a need for further serial testing based on the ESC zero/two-hour pathway guidelines. According to the ESC zero/two-hour algorithm, patients with an initial hs-cTn <3 ng/L are typically considered for immediate rule-out of NSTEMI, provided they have a low clinical probability and no dynamic ECG changes. Therefore, any subsequent hs-cTn testing in this specific subgroup was deemed unnecessary. This definition was applied retrospectively through a review of electronic patient records. Non-cardiac presentations were identified through a retrospective review of electronic patient records. Patients were categorised as having a non-cardiac presentation if their primary presenting complaint and clinical assessment did not include chest pain or other symptoms suggestive of acute coronary syndrome (e.g. dyspnea of suspected cardiac origin, syncope with a high suspicion of a cardiac cause). This included patients presenting with a wide range of other conditions, such as falls, delirium, or abdominal pain, where hs-cTn testing was ordered as part of a broader diagnostic workup. While there may have been some cases with overlapping symptoms, the primary reason for presentation was used to categorise patients. Staff confidence in using the ESC algorithm and awareness of assay-specific cut-offs were assessed through structured questionnaires administered to clinical staff before and after the educational interventions.

Data analysis

Data were extracted from the hospital’s electronic pathology and clinical records. Descriptive statistics were used to compare performance between cohorts. Comparisons between pre-implementation and post-implementation cohorts were performed for continuous and categorical variables. Continuous variables were expressed as mean ± standard deviation (SD) and compared using independent-samples t-tests. Categorical variables were compared using two-proportion z-tests, and Fisher’s exact test was also performed to confirm statistical significance. For each outcome, the mean or proportion difference between the cohorts was calculated, along with the corresponding 95% confidence intervals (CIs) for the difference. All statistical tests were two-tailed, with significance defined as p-values <0.05. Changes in adherence rates, unnecessary repeat testing, and inappropriate test ordering were specifically analysed as indicators of improvement [10]. All statistical analyses were performed using SPSS® Statistics version 28 software (IBM Corp., Armonk, NY, USA). The 95% confidence intervals (CIs) reported in our manuscript were calculated for the absolute differences between the pre-implementation and post-implementation cohorts. For continuous variables (e.g. time to first hs-cTn, zero to two-hour testing interval, length of stay), the CIs represent the 95% CI for the mean difference between the two cohorts. For categorical variables (e.g., adherence rates, documentation of ESC pathway, staff confidence), the CIs represent the 95% CI for the difference in proportions (percentage points) between the two cohorts. We acknowledge that our study involved multiple comparisons, which can increase the risk of Type I error. However, we did not make formal adjustments for multiple comparisons (e.g. Bonferroni correction) for the following reasons: (1) This was a real-world implementation study, and our analyses were primarily exploratory, aiming to identify areas of improvement following the intervention. In this context, a strict correction for multiple comparisons can be overly conservative and may obscure clinically meaningful findings. (2) The improvements were consistent and statistically significant across a wide range of related outcomes (e.g. adherence, efficiency metrics, staff knowledge), which provides strong evidence for a true intervention effect rather than random chance. (3) The p-values for our key findings were extremely low (many p < 0.0001), and the effect sizes were large. These results would remain highly significant even after applying a conservative correction for multiple comparisons. We did not encounter significant issues with missing or incomplete data for the primary and secondary outcomes reported in the study. The data for hs-cTn results, timing of tests, length of stay, and NSTEMI diagnoses were extracted from the hospital’s electronic pathology and clinical records, which are comprehensive and well-maintained. For the staff surveys, the response rates were high (approximately 85%), minimising the risk of significant bias from non-responders.

## Results

In the pre-implementation cohort, a total of 595 patients who presented to the SDEC received an hs-cTn test in November 2024. Of these, only 126 (21.1%) patients presented with chest pain. The rest of the cohort did not have symptoms suggestive of acute coronary syndrome, and this was considered inappropriate testing. Initial hs-cTn levels were distributed, as shown in Table 2.

**Table 2:** Distribution of initial hs-cTn results among patients attending SDEC in November 2024 (pre-implementation cohort). hs-cTn: high-sensitivity cardiac troponin; SDEC: Same-Day Emergency Care

Level	Number/Proportion
Very low (<3 ng/L)	383 (64.3%)
Low (3–119 ng/L)	202 (33.9%)
High (≥120 ng/L)	10 (1.6%)

**Figure 2:**
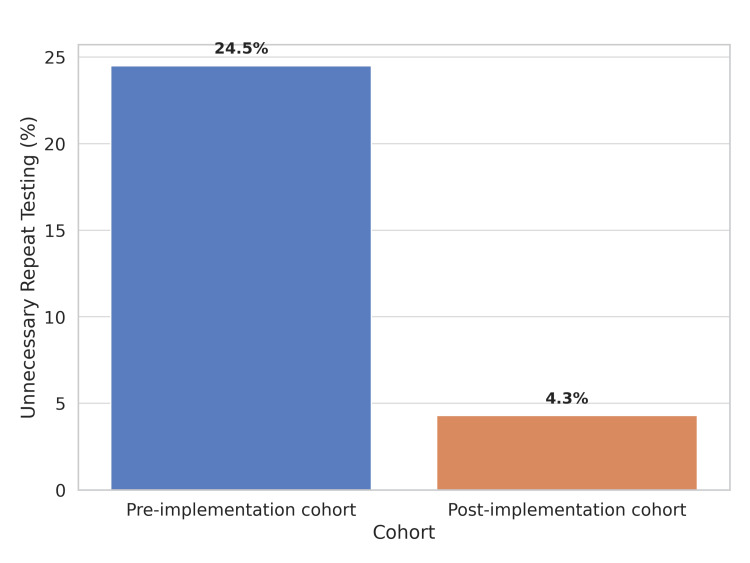
Unnecessary repeat hs-cTn testing for very low initial results in the pre-implementation and post-implementation cohort. hs-cTn: high-sensitivity cardiac troponin

Repeat testing was performed in 126 (21.1% of the total tested) patients. Among chest pain patients with very low initial troponin, 31 out of 126 (24.5%) underwent unnecessary repeat testing (Figure 2). One patient with low troponin had no repeat testing and was later diagnosed with myocardial infarction. Overall adherence to the ESC 0/2-hour algorithm was 74.2% (441/595 patients).

In the post-implementation cohort, following educational and process-based interventions, 340 patients had hs-cTn testing in June 2025. Among these, 89 (26.1%) patients had chest pain. The rest did not have symptoms suggestive of acute coronary syndrome, and this was considered inappropriate testing. Troponin distribution is presented in Table 3.

**Table 3:** Distribution of initial hs-cTn results among patients attending SDEC in June 2025 (post-implementation cohort) following educational and process-based interventions. hs-cTn: high-sensitivity cardiac troponin; SDEC: Same-Day Emergency Care

Level	Number/Proportion
Very low (<3 ng/L)	149 (43.8%)
Low (3–119 ng/L)	184 (54.1%)
High (≥120 ng/L)	7 (2%)

Repeat testing was performed in 28 (8.2% of the total) patients. Among chest pain patients with very low initial troponin, only 4 out of 89 (4.3%) received unnecessary repeat testing (Figure 2). No missed cases of myocardial infarction were identified. Overall adherence to the ESC algorithm improved to 94.4% (84/89 chest pain patients).

A comparison between pre- and post-implementation cohorts showed statistically significant improvements in multiple metrics (Table 4). The mean time from presentation to first hs-cTn decreased from 32.25 ± 4.17 minutes to 18.78 ± 6.32 minutes (mean difference = 13.47 minutes; 95% CI = 12.72-14.22; p < 0.0001). The interval between zero-hour and two-hour hs-cTn fell from 241.8 ± 15.43 minutes to 108.0 ± 6.14 minutes (mean difference = 133.80 minutes; 95% CI = 132.40-135.20; p < 0.0001). Length of stay in SDEC was reduced from 428 ± 17.34 minutes to 115 ± 10.56 minutes (mean difference = 313.00 minutes; 95% CI = 311.21-314.79; p < 0.0001).

**Table 4:** Comparison of mean time intervals and length of stay in SDEC between the pre-implementation and post-implementation cohort. P-values <0.05 were considered statistically significant. Statistical tests used: independent-samples t-test. hs-cTn: high-sensitivity cardiac troponin; SDEC: Same-Day Emergency Care; CI: confidence interval

	Pre-implementation, mean	Post-implementation, mean	Mean difference	95% CI (difference)	t-value	P-value
Time from presentation to first hs-cTn (minutes)	32.25	18.78	13.47	12.72–14.22	35.20	<0.001
Time between zero-hour and two-hour hs-cTn (minutes)	241.8	108.0	133.80	132.40–135.20	187.32	<0.001
Length of stay in SDEC (minutes)	428.0	115.0	313.00	311.21–314.79	342.72	<0.001

In addition, as shown in Table 5, there were large relative increases in documentation of the ESC algorithm pathway (1.0% vs. 74.1%; difference = 73.1 percentage points; 95% CI = 68.4-77.8; p < 0.0001) and the use of the risk stratification tool, the standard HEART score (1.0% vs. 62.8%; difference = 61.8 pp; 95% CI = 56.6-67.0; p < 0.0001). Ordering hs-cTn without prior ECG dropped from 98.1% to 16.7% (difference = 81.4 pp; 95% CI = 77.3-85.5; p < 0.0001). Non-cardiac symptom cases with elevated troponin fell from 33.9% to 8.0% (difference = 25.9 pp; 95% CI = 21.1-30.7; p < 0.0001).

**Table 5:** : Comparison of clinical practice measures, staff knowledge, and adherence to the ESC zero/two-hour hs-cTn algorithm between pre-implementation and post-implementation cohorts. P-values <0.05 were considered statistically significant. Statistical tests used: two-proportion z-test, Fisher’s exact test. ESC: European Society of Cardiology; hs-cTn: high-sensitivity cardiac troponin; CI: confidence interval; HEART: History, Electrocardiogram, Age, Risk factors, and Troponin

	Pre-implementation proportion	Post-implementation proportion	Difference	95% CI (difference)	Z-value (pooled SE)	P-value
Documentation of ESC algorithm pathway used	0.010	0.741	0.731	0.684–0.778	24.06	<0.001
Use of risk stratification scores (HEART Score)	0.010	0.628	0.618	0.566–0.670	21.45	<0.001
Ordering hs-cTn tests without ECG before blood sampling	0.981	0.167	0.814	0.773–0.855	25.78	<0.001
Elevated troponin results (low/high) in non-cardiac presentation	0.339	0.080	0.259	0.211–0.307	8.86	<0.001
Staff confidence in using the ESC algorithm	0.562	0.883	0.321	0.268–0.374	10.11	<0.001
Awareness of assay-specific cut-offs	0.000	0.816	0.816	0.775–0.857	26.27	<0.001
Adherence to the ESC two/two-hour algorithm	0.742	0.944	0.202	0.159–0.245	7.66	<0.001

There was no statistically significant difference in NSTEMI diagnosis among troponin-positive patients (4.3% vs. 3.2%; p = 0.403) or in 30-day MACE among patients ruled out by the algorithm (0.4% vs. 0.0%; p = 0.243).

**Figure 3:**
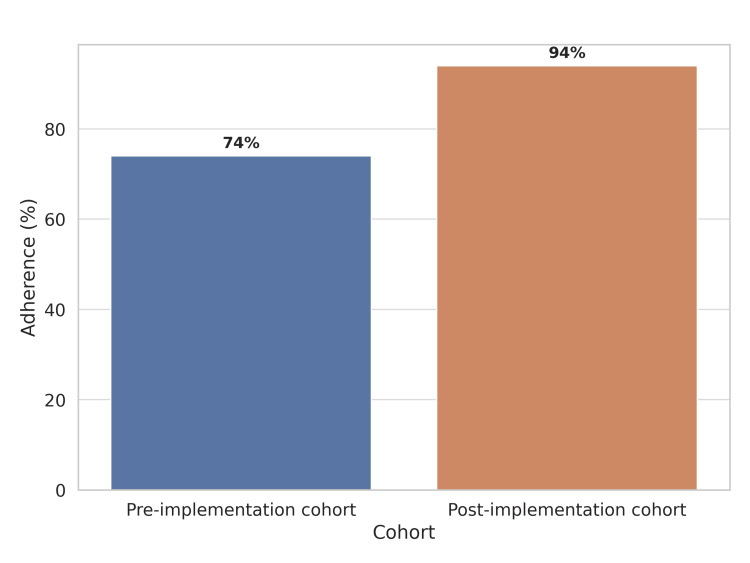
Adherence to the ESC zero/two-hour hs-cTn algorithm in the pre-implementation and post-implementation cohort. ESC: European Society of Cardiology; hs-cTn: high-senistivity cardiac troponin

Staff confidence in using the ESC algorithm improved from 56.2% to 88.3% (difference = 32.1 pp; 95% CI = 26.8-37.4; p < 0.0001), and awareness of assay-specific cut-offs rose from 0.0% to 81.6% (difference = 81.6 pp; 95% CI = 77.5-85.7; p < 0.0001). In addition, adherence to the ESC zero/two-hour algorithm increased from 74.2% to 94.4% (difference = 20.2 pp; 95% CI = 15.9-24.5; p < 0.0001) (Figure 3). Fisher’s exact test was also performed, yielding consistent significance for all comparisons (p < 0.0001).

## Discussion

In our study, we observed significant improvements across operational, process, and staff-reported metrics following the adoption of the ESC zero/two-hour hs-cTn pathway accompanied by structured educational interventions. Key outcomes included reductions in time from presentation to first hs-cTn (mean difference = 13.47 minutes; 95% CI = 12.72-14.22; p < 0.0001), interval between zero-hour and two-hour hs-cTn (mean difference = 133.8 minutes; 95% CI = 132.40-135.20; p < 0.0001), and length of stay in SDEC (mean difference = 313.0 minutes; 95% CI = 311.21-314.79; p < 0.0001). These substantial reductions align with systematic reviews demonstrating that shorter hs-cTn-based accelerated diagnostic protocols (ADPs), such as zero/one- or zero/two-hour strategies, are associated with reduced emergency department (ED) length of stay and increased early discharges without a rise in 30-day MACE [11,12].

Our magnitude of operational improvement is consistent with prior observational and real-world implementation studies. For instance, the RAPID-CPU study showed that implementing the ESC zero/one-hour protocol reduced median ED stay and increased discharge rates from 53.9% to 62.8%, with no excess mortality at 30 days [13]. Additional systematic reviews and meta-analyses reinforce that shorter-duration ADPs yield faster disposition and higher discharge rates compared to longer protocols, without compromising safety. While our study focused on the zero/two-hour pathway, the magnitude of improvement we observed in terms of reduced length of stay (mean difference of 313 minutes) is comparable to, and in some cases exceeds, the improvements reported in studies of the zero/one-hour pathway (e.g. the RAPID-CPU study, which showed a reduction in median ED stay from 5.3 hours to as low as 2.9-3.2 hours). This suggests that a well-implemented zero/two-hour pathway can achieve similar efficiency gains to the zero/one-hour pathway, making it a viable alternative in settings where the logistics of a one-hour repeat test are challenging [11,12,14].

Notably, our study also documents large improvements in documentation of the ESC algorithm increased from 1.0% to 74.1%, and HEART score use increased from 1.0% to 62.8% (all p < 0.0001). Implementation literature underscores that these gains are most likely when assay roll-out is supported by formal education, protocol standardisation, and integration of risk scores into clinical workflow [15]. Centres that pair hs-cTn implementation with structured training achieve more sustainable improvements in appropriate testing and algorithm adherence [15].

To contextualise the magnitude of our observed changes beyond statistical significance, we provide effect size interpretations for our key findings as follows; the improvement from 74.2% to 94.4% in the adherence to ESC zero/two-hour algorithm represents a large effect size (Cohen’s h = 0.54), indicating a substantial and clinically meaningful improvement in practice, the mean difference of 133.8 minutes between zero-hour and two-hour hs-cTn represents a very large effect size (Cohen’s d > 2.0), signifying a dramatic improvement in diagnostic efficiency, and the mean difference of 313.0 minutes in the length of stay also represents a very large effect size (Cohen’s d > 2.0), highlighting a significant impact on patient flow and resource utilization.

The single missed myocardial infarction case identified in the pre-implementation cohort was indeed considered in our safety outcome analyses. This case, where a patient with a low troponin level had no repeat testing and was later diagnosed with myocardial infarction, underscores the importance of adherence to standardised pathways. Safety outcomes in the post-implementation cohort were reassuring. We observed no increase in 30-day MACE among patients ruled out via the pathway. Moreover, there were no missed cases of myocardial infarction identified in the post-implementation cohort following the implementation of the ESC zero/two-hour algorithm, and NSTEMI diagnosis rates among troponin-positive patients remained stable. This parallels findings from other large datasets demonstrating that hs-cTn-based ADPs preserve short-term safety while enabling earlier discharge [12,15,16]. However, ensuring patient safety depends heavily on strict adherence to assay-specific cut-offs, standardised ECG assessment, and robust governance, all areas in which our interventions provided improvements [15].

Once again, the reductions in repeat or inappropriate testing and improved documentation likely enhanced laboratory efficiency and patient throughput, findings similar to those reported in implementation studies where hs-cTn adoption resulted in lower testing volumes and fewer admissions [17]. Similarly, our observed rise in staff confidence and assay-awareness reflects that targeted education can improve fidelity to new protocols [15]. Although our NSTEMI detection rate did not significantly change, the literature suggests that diagnostic yield is influenced by pretest probability, delta thresholds, and integration of risk scores, areas where future local calibration may be beneficial [15]. The standard HEART score was interpreted in conjunction with hs-cTn values and the broader clinical context, in line with validated approaches described in the literature. The integration of structured risk stratification with high-sensitivity troponin assays has been shown to enhance diagnostic accuracy, reduce unnecessary admissions, and support safe early discharge in appropriately selected patients [18].

However, one persistent area of concern is the high proportion of hs-cTn testing in patients without chest pain. In both cohorts, over 70% of tests were ordered for non-cardiac presentations, suggesting that troponin is often used as a general screening tool in acutely unwell patients. This practice can reduce diagnostic specificity and increase the likelihood of false-positive results, leading to unnecessary investigations and admissions [7,8]. This underscores a critical area for future research and intervention. We strongly advocate for further evaluation of targeted interventions specifically designed to address this issue. There will also be a need to investigate the potential reasons for the persistent overuse of hs-cTn in non-cardiac presentations, which may include defensive medicine practice, lack of clear guidance for non-cardiac presentations, and system-level factors.

We acknowledge several limitations that may affect the generalizability and interpretation of our findings. First, this was a single-centre study conducted in a UK district general hospital. The specific patient population, organisational structure, and local practices may limit the direct transferability of these results to other healthcare settings with different resources or patient demographics. Second, the retrospective pre-post observational design, while practical for evaluating real-world implementation, is susceptible to confounding factors and temporal trends that may not have been fully accounted for. Although interventions were targeted, other concurrent changes in clinical practice or patient presentation patterns could have influenced the outcomes. We addressed these potential confounders through several considerations: (1) the primary aim was to evaluate the impact of specific, targeted interventions; (2) educational interventions were comprehensive, targeting all relevant staff groups; (3) the specific outcomes measured were primarily influenced by the implemented protocol and staff training rather than seasonal fluctuations; and (4) the emphasis was on demonstrating the effectiveness of the intervention within our specific institutional context. Third, while the study demonstrated significant improvements in adherence and operational efficiency, it did not comprehensively evaluate long-term clinical outcomes such as one-year mortality, re-hospitalisation rates, or quality of life. The absence of these long-term data points limits the full assessment of patient benefit. Finally, despite significant improvements, the study highlighted a persistent issue of hs-cTn requests for non-cardiac presentations (>70% of requests), indicating that while the pathway improved appropriate use for suspected cardiac chest pain, broader education on appropriate indications for troponin testing remains a challenge and affects the overall efficiency gains [15].

To sustain and further enhance the long-term success of the ESC zero/two-hour hs-cTn pathway implementation, we propose several key next steps and measurable goals. These include implementing a continuous education program for all relevant clinical staff, with a measurable goal of achieving >95% staff competency in algorithm application through annual assessments. We also plan to integrate the ESC zero/two-hour algorithm directly into the hospital’s electronic health records system, aiming to reduce inappropriate hs-cTn ordering for non-cardiac presentations by an additional 25% within 12 months post-electronic algorithm integration. Furthermore, establishing a routine audit and feedback mechanism, providing monthly reports to clinical departments, will aim to maintain adherence to the ESC zero/two-hour pathway at >90% consistently over 24 months.

The observed improvements in diagnostic efficiency and reduced length of stay have significant cost and efficiency implications, underscoring the practical value of this intervention. The mean reduction of 313 minutes (approximately 5.2 hours) in SDEC length of stay per patient translates into substantial savings in bed utilisation and increased capacity. For a high-volume unit, this can free up numerous bed-days annually, allowing for more efficient patient flow and reduced overcrowding. Moreover, faster diagnostic turnaround times and reduced unnecessary repeat testing lead to more efficient use of laboratory resources, nursing time, and physician time, minimising waste. While not directly measured, a more confident and rapid rule-out of NSTEMI can potentially reduce unnecessary hospital admissions, further contributing to cost savings and improved patient experience. These efficiencies collectively contribute to a more sustainable healthcare system by optimising resource allocation and reducing operational costs associated with prolonged hospital stays and inefficient diagnostic processes.

Our findings support the wider adoption of hs-cTn-enabled ADPs combined with standardised risk scoring and structured education. They highlight that operational efficiencies and improved clinical adherence can be achieved without compromising safety when implementation addresses documentation, ECG completion, and assay-specific thresholds. Future research should include multicentre, patient-level evaluations comparing zero/one vs. zero/two-hour algorithms in real-world settings, cost-effectiveness assessments (covering lab workload, admissions, resource utilisation), and inclusion of patient-reported outcomes to assess satisfaction and experience, areas that remain underexplored but are of increasing importance [1,6].

## Conclusions

This study demonstrated that structured implementation of the ESC zero/two-hour hs-cTn pathway substantially improved diagnostic efficiency, adherence, and safety without increasing adverse outcomes. Moreover, simple, targeted interventions, such as educational sessions, visual algorithm prompts, and multidisciplinary collaboration, can significantly improve adherence to evidence-based troponin testing protocols. While the project succeeded in improving algorithm compliance, it also highlighted persistent overuse of troponin testing in patients without chest pain, underscoring the need for further upstream triage education and system-level change. The observed improvements in diagnostic speed and reduced length of stay also translate into significant operational benefits, including increased bed availability and improved patient flow, without compromising patient safety. Sustained improvement will require integration of structured risk stratification tools and continued education for new clinical staff. Future efforts will focus on digital integration and continuous audit-feedback systems to reinforce adherence and address inappropriate troponin use in non-cardiac presentations. A longer-term follow-up study at one year will be necessary to assess the sustainability of these improvements and potential for attrition.

## References

[1] Goodacre S, Cross E, Arnold J, Angelini K, Capewell S, Nicholl J (2005). The health care burden of acute chest pain. Heart.

[2] Thygesen K, Alpert JS, Jaffe AS, Chaitman BR, Bax JJ, Morrow DA, White HD (2019). Fourth universal definition of myocardial infarction (2018). Eur Heart J.

[3] Byrne RA, Rossello X, Coughlan JJ (2023). 2023 ESC Guidelines for the management of acute coronary syndromes. Eur Heart J.

[4] Lowry MT, Anand A, Mills NL (2021). Implementing an early rule-out pathway for acute myocardial infarction in clinical practice. Heart.

[5] Chapman AR, Bularga A, Mills NL (2020). High-sensitivity cardiac troponin can be an ally in the fight against COVID-19. Circulation.

[6] Carlton EW, Than M, Cullen L, Khattab A, Greaves K (2015). 'Chest pain typicality' in suspected acute coronary syndromes and the impact of clinical experience. Am J Med.

[7] Sandoval Y, Smith SW, Thordsen SE (2017). Diagnostic performance of high sensitivity compared with contemporary cardiac troponin I for the diagnosis of acute myocardial infarction. Clin Chem.

[8] Lee KK, Ferry AV, Anand A (2019). Sex-specific thresholds of high-sensitivity troponin in patients with suspected acute coronary syndrome. J Am Coll Cardiol.

[9] Bevins NJ, Chae H, Hubbard JA, Castillo EM, Tolia VM, Daniels LB, Fitzgerald RL (2022). Emergency department management of chest pain with a high-sensitivity troponin-enabled 0/1-hour rule-out algorithm. Am J Clin Pathol.

[10] Sadowski BW, Lane AB, Wood SM, Robinson SL, Kim CH (2017). High-value, cost-conscious care: iterative systems-based interventions to reduce unnecessary laboratory testing. Am J Med.

[11] Hill J, Essel NO, Yang EH, Dennett L, Rowe BH (2024). Effectiveness of accelerated diagnostic protocols for reducing emergency department length of stay in patients presenting with chest pain: a systematic review and meta-analysis. PLoS One.

[12] Hsiao JJ, Celedon MA, Rudolph JL (2024). Accelerated diagnostic protocols using high-sensitivity troponin assays to rule in or out myocardial infarction: a systematic review. JEM Rep.

[13] Stoyanov KM, Hund H, Biener M (2020). RAPID-CPU: a prospective study on implementation of the ESC 0/1-hour algorithm and safety of discharge after rule-out of myocardial infarction. Eur Heart J Acute Cardiovasc Care.

[14] Westwood M, van Asselt T, Ramaekers B (2015). High-sensitivity troponin assays for the early rule-out or diagnosis of acute myocardial infarction in people with acute chest pain: a systematic review and cost-effectiveness analysis. Health Technol Assess.

[15] Bularga A, Lee KK, Stewart S (2019). High-sensitivity troponin and the application of risk stratification thresholds in patients with suspected acute coronary syndrome. Circulation.

[16] Chapman AR, Mills NL (2020). High-sensitivity cardiac troponin and the early rule out of myocardial infarction: time for action. Heart.

[17] Ford JS, Chaco E, Tancredi DJ, Mumma BE (2021). Impact of high-sensitivity cardiac troponin implementation on emergency department length of stay, testing, admissions, and diagnoses. Am J Emerg Med.

[18] Chapman AR, Hesse K, Andrews J (2018). High-sensitivity cardiac troponin I and clinical risk scores in patients with suspected acute coronary syndrome. Circulation.

